# Mediators and Moderators of Reinforced Self-Affirmation as a Method for Reducing the Memory Misinformation Effect

**DOI:** 10.3389/fpsyg.2021.666707

**Published:** 2021-11-23

**Authors:** Malwina Szpitalak, Romuald Polczyk

**Affiliations:** Institute of Psychology, Jagiellonian University, Kraków, Poland

**Keywords:** feedback, memory, misinformation effect, reducing suggestibility, reinforced self-affirmation, witness testimony

## Abstract

The misinformation effect occurs when an eyewitness includes information in his or her account that is incongruent with the event he or she witnessed, and stems from being exposed to incorrect external sources. This is a serious threat to the quality of witness testimony and to the correctness of decisions reached by courts. However, few methods have been developed to reduce the vulnerability of witnesses to misinformation. This article presents such a method, namely, reinforced self-affirmation (RSA), which, by increasing memory confidence of witnesses, makes them less inclined to rely on external sources of information and more on their own memory. The effectiveness of this method was confirmed in three experiments. It was also found that memory confidence, but not general self-confidence, is a mediator of the impact of RSA on misinformation effect (ME), and that contingent self-esteem and feedback acceptance, but not sense of self-efficacy or general self-esteem, are moderators of this impact. It is concluded that RSA may be a promising basis for constructing methods, which can be used by forensic psychologists in real forensic settings.

## Introduction

Misinformation of various kinds is very commonplace in our lives, and it is difficult to undo its influence (Walter and Murphy, 2018). Misinformation is also influential in the context of eyewitness testimony (Luna and Martín-Luengo, 2012). Given the dramatic effects, which distorted testimony can have on judicial decisions, including wrongful convictions and acquittals of real perpetrators, it is mandatory to construct methods that can make eyewitnesses more resistant to misinformation.

The present paper explores one such method: reinforced self-affirmation (RSA). This is a way of reducing the memory misinformation effect (ME), which consists in including testimony information, which does not stem from a given event but from other sources. ME is typically studied within a three-stage experimental paradigm (seminal research: Pezdek, 1977; Loftus et al., 1978) in which participants are first exposed to some original material. It can be a video clip (e.g., Cohen and Harnick, 1980), a series of slides (e.g., Loftus et al., 1978), an audio recording (e.g., Szpitalak and Polczyk, 2010), or a text to be read (e.g., Hertel et al., 1980). After some time, participants are exposed to post-event material; for example, they read a description of the origina material, which, in the experimental group, contains some information, which is inconsistent with the original event (e.g., Zaragoza and Lane, 1994), or they answer a series of questions, which, in the experimental group, contain some incorrect premises (e.g., Loftus et al., 1978). In some research, live confederates presented misinformation while interacting with the real participants (e.g., Hope et al., 2008; Mojtahedi et al., 2019a,b). Subsequently, the participants answer a series of questions about the original event, including critical questions relating to the misinformation. In almost all experiments of this kind, it has been found that participants in the experimental group perform worse on the final memory test as they usually include some of the misinformation in their answers (for a review, see Zaragoza et al., 2007).

Many theoretical explanations of the nature of ME for the misinformation effect have been proposed, starting with the classical theories stating that misinformation overwrites the original memory trace (Loftus, 1975) or, in a way, “integrates” into the original memory (Loftus et al., 1978). Another explanation, rooted in the activation-based memory model, stated that, as a result of the misinformation, there are *two* memory traces attached to the critical event, one for the original and one for the misleading information, and activation is shared by the traces, so either could be given as a response (Ayers and Reder, 1998). Another explanation was based on the retrieval-based explanation of forgetting and stated that original information and the misinformation coexist in memory, the latter making the former more difficult to retrieve (Bekerian and Bowers, 1983; Bowers and Bekerian, 1984). Yet another explanation was based on the fuzzy-trace theory and posited that false memories occur primarily because gist memories are falsely ascribed to experience (Reyna and Lloyd, 1997).

Nowadays, it seems that the most popular theoretical explanation of the misinformation effect is the source monitoring theory, which posits that the participants confuse information stemming from the postevent material with their real memories of the original event; in other words, they misattribute the source of their information (e.g., Lindsay and Johnson, 1989; Zaragoza and Koshmider, 1989; Zaragoza and Lane, 1994; Cann and Katz, 2005; Higham et al., 2011). One of the most sophisticated versions of the source-monitoring accounts, including the model Composite Holographic Associative Recall Model (CHARM), was presented by Dodhia and Metcalfe (1999). It explains source monitoring errors in terms of the implications of retrieving a superimposed representation that contains both the original events superimposed on the misleading suggestion (the van).

All the above-mentioned explanations of the misinformation effect share the core assumption that there is some kind of memory malfunction caused by misinformation. However, there is strong empirical evidence confirming that this is not necessarily the case, and people can give memory accounts consistent with misinformation even if there is nothing wrong with their memory. First of all, McCloskey and Zaragoza (1985) presented a strong theoretical and empirical case, showing that, for the misinformation effect to occur, it is enough that two fractions of participants are present: (1) those who, at the moment of the final memory test, do not remember the original information (for example, because it was never encoded) and/or (2) those who remember *both* the original and misleading information, and answer in accordance with the latter. McCloskey and Zaragoza (1985) presented their participants with two options in the final memory test: the one is consistent with the original information; the second one is inconsistent neither with the original nor with the postevent information (instead of an option consistent with misinformation). No differences were present between the misled and control conditions, which undermine any explanations of the misinformation effect that are based on the memory impairment hypothesis.

Moreover, there are experimental data directly confirming that ME can occur even if participants do remember the correct information from the original event but still give accounts consistent with the misinformation, probably due to lack of confidence in their own memories (Blank, 1998; Szpitalak and Polczyk, 2010; Polczyk, 2017). For example, in research by Polczyk (2017), the participants were administered the standard procedure for testing for the misinformation effect; *afterward*, they were debriefed and given full explanations about the procedure, and again asked what they saw in the original film and read in the postevent material. Many of those who gave answers consistent with misinformation were perfectly able to correctly indicate what was in both sources; thus, they yielded to misinformation in spite of being aware of the discrepancies between the original and postevent materials. In a broader sense, this is a manifestation of informational influence (Mojtahedi et al., 2019a,b).

Some participants yield to misinformation even if they are allowed to access the original and post-event sources while answering the questions from the final memory test; thus, they simply cannot misremember the content of the sources nor can they misattribute them (Polak et al., 2016).

As the basis for the present research, the theory by Blank (1998) was adopted, called an integrative framework for the analysis of memory and performance (I MP) in eyewitness suggestibility experiments. In short, I MP basically assumes that subjects taking part in experiments concerning the misinformation effect are facing a problem-solving process. When answering the questions on the final memory test, they have to find a solution to a memory task. The solution is based on memory states – available information in memory and on the internal representation of the memory task. In particular, this theory posits that there are participants who have information about the content and the source of the original event, as well as about the postevent material. Such participants are fully aware of the discrepancies between both sources. Provided that they assume consistency – they do not assume that they are deliberately misled – they may adopt different strategies to resolve the perceived discrepancies. In particular, some of them may answer in accordance with the postevent material, for example, because they do not trust their own memories.

There is surprisingly little research on the development of methods that aim to undo the suggestive influence of misinformation or to immunize against it, despite the fact that such research may be extremely useful in real forensic settings. One of the most often explored methods is simply warning the participants against possible discrepancies between the original and post-event materials (Greene et al., 1982). The efficacy of this method varied considerably; in some research, it was not effective at all (e.g., Greene et al., 1982; Neuschatz et al., 2001); in some others, it seemed to reduce the misinformation effect almost completely (e.g., Highhouse and Bottrill, 1995; Oeberst and Blank, 2012; see also the meta-analysis: Blank and Launay, 2014).

Apart from warning, not many other methods have been researched. In some research, a memory-enhancing technique, the cognitive interview reduced the vulnerability to misinformation among children (Holliday and Albon, 2004) and among elderly people (Holliday et al., 2012). A technique similar to the Cognitive Interview, Self-Administered Interview, also seemed to reduce the misinformation effect (Gabbert et al., 2012). However, such results were not present in research by Centofanti and Reece (2006).

As for other methods, English and Nielson (2010) found that triggering arousal reduced yielding to misinformation. Clifasefi et al. (2007) as well as Parker et al. (2008) showed that a placebo presented to participants as a substance that seemingly enhanced cognitive processes improved their ability to resist misinformation. However, this result was not replicated in research by Nastaj et al. (2019). Wagstaff et al. (2011) found that focused meditation reduced interrogative suggestibility (Gudjonsson, 1997), although no clear results were obtained in the case of the standard three-stage procedure. Another experiment suggested that horizontal saccadic eye movements (but not vertical ones) reduced susceptibility to misinformation in the three-stage paradigm (Parker et al., 2009), and Szpitalak and Polczyk (2014) showed that mental warm-up reduces this susceptibility, while mental fatigue increases it.

As can be seen, there were not many techniques developed for reducing the misinformation effect, and the existing ones were not explored extensively (apart from warning). Moreover, many of them (apart from warning and cognitive interview) are not applicable in real forensic settings; one cannot arouse real witnesses by presenting them with disturbing videos, giving them medicaments, or asking them to make eye movements or meditate. Therefore, an exploration of methods reducing the misinformation effect or undoing the effects of misinformation is still warranted. The present research aims at this direction by exploring one such method: RSA (Szpitalak, 2012).

The basic premise of RSA was the assumption that there is a proportion of participants, which, in fact, do remember the correct original information while performing the final memory test. We further assume that a proportion of such participants gives answers that are consistent with the external misinformation but are inconsistent with their own correct memory due to their lack of confidence in it (Blank, 1998; Van Bergen et al., 2010). It was, therefore, assumed (Szpitalak, 2012) that increased confidence in one’s own memories should decrease the tendency to rely on the post-event material in the case of participants who, in fact, are aware of the discrepancies between the original and post-event material but believe their memories regarding the former are wrong and, therefore, prefer to rely on the latter.

The idea that self-confidence may be beneficial in the context of eyewitness memory was based on existing data, which suggest that it is advantageous in various areas. For example, it seems to improve leader performance (Hollenbeck and Hall, 2004), results on reasoning tests (Beckmann et al., 2009), or even intelligence tests (Stankov and Crawford, 1997), other cognitive competences (Kleitman and Stankov, 2007), school achievement (Srivastava, 2013), or oral presentation competences (Al-Hebaish, 2012). Most interestingly, self-confidence proved to be a predictor of reliance on oneself as a source of information (Barber, 2008), and of resisting social pressure (MacBride and Tuddenham, 1965). Also, there is research suggesting a direct link between self-confidence and resistance to suggestion in the context of witness testimony (Vrij and Bush, 2000) and memory conformity (Thorley and Kumar, 2017).

Reinforced self-affirmation is based on two elements: self-affirmation and positive feedback on memory functioning. Self-affirmation is induced by means of having participants write down their greatest achievements in life (see the detailed description in the method below). Such a method has proved effective in inducing self-affirmation in existing research (Schimel et al., 2004). In turn, a positive impact of self-affirmation on self-confidence was also found in research experiments (Petruzzello and Corbin, 1988; Compte and Postlewaite, 2004; Sherman and Cohen, 2006; Takai, 2011). As for positive feedback, there is research suggesting that it increases self-confidence (McCarthy, 1986; Fishbach et al., 2010) and reduces interrogative suggestibility (Tata and Gudjonsson, 1990). In sum, both self-affirmation and positive feedback are promising methods of increasing self-confidence, which, in turn, is expected to reduce the tendency to rely on external sources and, instead, to give reports based on one’s own memories.

The efficacy of RSA in reducing ME has been repeatedly confirmed and replicated (Szpitalak, 2012; Szpitalak and Polczyk, 2013, 2015b,2019a,b). The aim of the present paper is to further replicate its efficacy and provide data concerning the possible mechanisms of its impact. Therefore, the first hypothesis is that RSA will reduce ME. Additionally, some mediators and moderators of this main effect will be studied.

First of all, the main hypothesis concerning RSA is the assumption that it increases self-confidence, which, in turn, results in an enhanced tendency to rely on one own’s memories instead of information included in post-event material. If this is so, then a mediation should be present: RSA should affect ME *via* increased self-confidence. Moreover, as the task included in the ME procedure concerns memory, and feedback in RSA also concerns memory, it can be expected that, especially, self-confidence related to memory is involved. Therefore, it was hypothesized that this mediation will be present in the case of memory-related self-confidence but not in the case of general self-confidence.

Some moderators of the impact of RSA on ME were also analyzed. The first one was self-esteem. Individuals with high self-esteem might already have access to a wide range of positive self-feelings (Steele et al., 1993; Dodgson and Wood, 1998; Sherman and Cohen, 2006; Pietersma and Dijkstra, 2012). As such, RSA might confer little advantage to these individuals in terms of encouraging them to rely on their own memory. By contrast, individuals with low self-esteem might have a more limited array of positive self-feelings that are readily available to them when faced with threatening information. Accordingly, an explicit self-affirmation manipulation might provide an important means of boosting self-esteem for these individuals (see also Spencer et al., 2001; Düring and Jessop, 2015). Thus, self-esteem may be a moderator of the impact of RSA on ME.

The term “contingent self-esteem” (Deci and Ryan, 1995) seems to be very useful, too. Contingent self-esteem “…refers to feelings about oneself that result from – indeed, are dependent on matching some standard of excellence or living up to some interpersonal or intrapsychic expectations” (Deci and Ryan, 1995, p. 32). Contingent self-esteem is dependent on matching standards and is directly linked and dependent on perceived successes and failures (Kernis et al., 1993; Park et al., 2004). Therefore, people with contingent self-esteem should be particularly prone to procedures that aim to increase self-confidence, like RSA.

In light of these considerations, it seems that both the level of self-esteem and its stability should moderate the impact of RSA on ME. Persons with stable, reinforcement-independent self-esteem may be less susceptible to RSA than those with contingent self-esteem, which is dependent on external feedback. It was, therefore, hypothesized that RSA would mainly be effective among participants with contingent self-esteem. Also, it was postulated that RSA would be more effective in the case of low general self-esteem because people with high self-esteem may benefit from RSA less – they are probably already self-confident, and efforts to additionally increase this self-confidence may be less effective.

In a very similar vein, a second moderator was postulated, namely, sense of self-efficacy. Self-efficacy (Schwarzer, 1993) refers to self-perceived general efficacy in coping with various tasks and achieving goals. It was assumed that people who perceive their self-efficacy as high would benefit from increased self-confidence less than those whose sense of self-efficacy is low. It should be so because people who feel that they are effective across a range of tasks and goals may be more relying on themselves and perhaps having higher and more stable self-esteem. In the case of such people, increasing self-confidence may not be particularly effective as this self-confidence is probably already relatively high. In contrast, people perceiving their self-efficacy as low may tend to have lower self-confidence and, in turn, benefit from RSA more. In sum, this would cause self-efficacy to be a moderator of the impact of RSA on ME.

The third moderator analyzed in the present study is acceptance of positive feedback. Ilgen et al. (1979) defined feedback acceptance as “the recipient’s belief that the feedback is an accurate portrayal of his or her performance” (p. 356). There are examples of experiments on positive feedback, which show that its acceptance is, by no means, universal and guaranteed and that such acceptance may influence the results obtained. For example, it was found that the efficacy of feedback when avoiding “harmful” food proved dependent on its acceptance (Scoboria et al., 2008; Bernstein et al., 2011; Mantonakis et al., 2013). Similarly, Anseel et al. (2009) showed that feedback acceptance influences attitude change. In the present research, it was hypothesized that feedback acceptance would moderate the impact of RSA on ME; this impact will be higher for the participants who believed the feedback.

Three experiments were performed. In each one, the existence of the misinformation effect and the efficacy of RSA were analyzed. In addition, in Experiment 1, memory confidence and general confidence were analyzed as mediators of the impact of RSA on yielding to misinformation. In Experiment 2, both these mediators were analyzed again, and contingent and general self-esteem, as well as the sense of self-efficacy, was studied as a possible moderator. In Experiment 3, memory confidence was analyzed as the mediator, and the efficacy of feedback in RSA, as a possible moderator.

## Power and Sample Size Considerations

Power and sample size analysis was performed by means of the software G*POWER 3.1.9 (Faul et al., 2007). The required sample size was calculated for the power 95% for three effect sizes commonly assumed in such analysis, namely, Cohen f = 0.10 (small effect), 0.25 (medium), and 0.40 (large). Denominator *df* = 1 and four groups were assumed (the design in all three experiments was 2 × 2, see description below). For the main effects, as well as the interaction, the necessary sample sizes were 1,302, 210, and 84, respectively. Given the resources available, a sample size of about 210 was assumed, sufficient to detect medium and large effects, but the small one. In Experiments 2 and 3, the sample size in the experimental misled groups was increased, as these experiments focused on hypotheses, which could only be analyzed in the misled groups.

## General Strategy of Analyzing the Data

In each of the three experiments, there were three general aims: (1) to replicate the misinformation effect; (2) to replicate the efficacy of RSA; and (3) to explore mediators and moderators of the impact of RSA on yielding to misinformation. The first aim was analyzed by means of the main effect of misinformation in the analysis of variance and the second one by analyzing the interaction between misinformation and RSA and appropriate simple main effects, following the existing guidelines (Rosenthal and Rosnow, 1985; Rosnow and Rosenthal, 1989). The analyses concerning the third aim were performed in the group of misled participants only. The number of answers consistent with misinformation was the dependent variable, reflecting the individual susceptibility to misinformation. RSA was the predictor, and mediators and moderators of its impact on yielding to misinformation were analyzed.

## Experiment 1

The aims of Experiment 1 were to confirm the mediating effect of self-confidence in the impact of RSA on yielding to misinformation and to replicate the misinformation effect itself. Also, it was expected that self-confidence related to memory would be a statistically significant mediator of the impact of RSA on yielding to misinformation, whereas general self-confidence would not. The latter hypothesis will be tested with the Bayesian approach as it is difficult to prove the non-existence of effects with classical NHST methodology.

The following hypotheses were tested in Experiment 1:

1. The misinformation effect will be present: the number of answers consistent with misinformation will be higher for misinformed participants than for those in the control condition.
2. RSA will be effective: the number of answers consistent with misinformation will be lower for misinformed participants who undergo the RSA procedure than for those who do not.
3. Memory confidence will be a mediator of the impact of RSA on the misinformation effect.
4. General self-confidence will not be a mediator of the impact of RSA on the misinformation effect.

### Method – Experiment 1

#### Participants

Two hundred and thirteen subjects took part in the experiment (125 women, 88 men). Their mean age was 17.4 years (SD = 0.8; range: 15–19). The experiment took place during school classes. Various schools were chosen randomly; none of them included participants of previous studies on the misinformation effect (and different schools were chosen for the three experiments). No compensation was given for participation. The consent of the parents was not collected – it was not required in the schools. Two participants failed to complete the memory confidence questionnaire, and one participant failed to complete the general confidence questionnaire.

#### Materials

The study used a 2- and 1/2-min audio recording of some seemingly planned higher education reforms, prepared by the authors and recorded by a professional actor. These materials have been successfully used in other studies (Szpitalak, 2012). The post-event material was a written description summarizing the audio recording; in the misinformed group, it contained 10 details that were different from or additional to the original material. The final memory test consisted of 19 forced-choice questions; 10 of which were critical: the participants had to choose between the correct option or the option consistent with the misinformation in the form of a Yes/No test. Additionally, a short questionnaire, created by the authors of this study, was applied to measure memory confidence and general self-confidence. It consisted of five questions relating to the current quality of memory, and another five relating to self-confidence, e.g., “At the moment, I am assessing my memory”; “I am assessing my self-confidence at the moment:” (This questionnaire and all other materials are provided in Supplementary Material). Answers were given on a 7-point Likert-like scale, from 1: very low to 7: very high.

#### Procedure

The experiment was conducted during school classes. The participants were told that its purpose was to check opinions of students about the planned reforms in higher education. At the beginning, the participants listened to an audio recording (original material). In the recording, a single person advertises the new reform (see Supplementary Material). The instruction asked the participants to listen carefully, without giving additional information. After that, they were asked to answer a few questions about what they thought of the reform proposals that had been presented to them; this was done to support the cover story and was adopted from similar research by Apsler and Sears (1968). The questions did not relate to critical items. Then, after about 12 min, during which filler questionnaires were applied, the participants, under the pretext of refreshing the content of their memories, read the post-event material. Immediately after this, the RSA procedure took place. The first part aimed at inducing self-affirmation. The participants in the RSA group were asked to write down their greatest life achievements, while the other half (the control group) were asked to describe their ways home from school. Afterward, faked positive feedback on their memory quality was provided: all the participants were given a surprise memory task consisting in memorizing as many nouns as possible from a list of 60 nouns in a time period of 2 min. After these 2 min, the lists were removed, and the participants were asked to write down all the nouns they could remember. In the RSA group, the participants wrote the nouns in numbered slots so that they knew exactly how many nouns they were able to remember. In the control group, the slots were not numbered. Next, in the RSA group, the participants were told the “average mean number of nouns usually remembered.” This number was false; it was approximately 1.5 SD lower than the real average. In this way, most participants in this group “learned” that their memory was better than average. In the control group, no feedback was provided. In the next stage, the participants completed a questionnaire to check their general and specific self-confidence regarding the functioning of their memory in order to verify the efficacy of RSA. Next, the final memory test concerning the original material was administered in order to analyze the misinformation effect. At the end, the participants were debriefed.

Thus, the experimental design included two between-subjects factors: misinformation (no misinformation or misinformation present) × RSA (present or absent).

#### Results and Discussion – Experiment 1

In order to verify the efficacy of RSA, the differences between the groups in which it was applied as compared to the control groups were analyzed, with memory confidence and general self-confidence as dependent variables. Memory confidence was significantly higher in the RSA group as compared with the control group [*M* = 4.83, SD = 1.44 vs. *M* = 4.12, SD = 1.22; *F*(1,209) = 14.49, *p* < 0.001, η^2^ = 0.07]. In the case of general self-confidence, no significant effect of RSA was present [*M* = 4.53, SD = 1.11 vs. *M* = 4.47, SD = 0.83; *F*(1,210) = 0.20, *p* = 0.653, η^2^ < 0.01]. This confirms the efficacy of RSA in the case of memory-related self-confidence.

**FIGURE 1:**
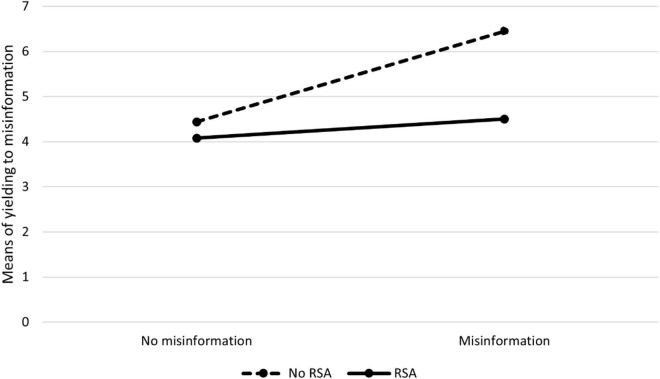
Means of yielding to misinformation across experimental conditions in Experiment 1.

Descriptive results across experimental conditions are presented in Table 1 and Figure 1.

**TABLE 1:** Means (SDs, number of participants) of yielding to misinformation across experimental conditions in Experiment 1.

RSA	No misinformation	Misinformation	Total
Absent	4.44 (1.50, 50)	6.45 (2.11, 51)	5.46 (2.09, 101)
Present	4.08 (1.82, 52)	4.50 (2.59, 60)	4.30 (2.27, 112)
Total	4.25 (1.68, 102)	5.40 (2.57, 111)	4.85 (2.25, 213)

A 2 × 2 analysis of variance revealed a significant main effect of misinformation, with misinformed participants giving more answers consistent with the misinformation [*F*(1,209) = 18.23, *p* < 0.001, η^2^ = 0.08]. This confirms Hypothesis 1, which concerns the presence of the misinformation effect. The main effect of RSA was also significant, with the participants in the RSA group giving less answers consistent with the misinformation [*F*(1,209) = 16.48, *p* < 0.001, η^2^ = 0.07]. The interaction of the misinformation factor with RSA was also significant [*F*(1,209) = 7.76, *p* = 0.006, η^2^ = 0.04], and the inspection of relevant means in Table 1 suggests that that the fall in the mean number of answers consistent with the misinformation was greater in the misinformed group than in the control group. Indeed, analysis of simple effects confirmed that there was no significant difference between the RSA and no-RSA groups in the condition without misinformation [*F*(1,209) = 0.78, *p* = 0.378, η^2^ < 0.01]. This makes sense as RSA is directed and expected to be effective only in the group of misinformed participants. In this group, its impact was significant [*F*(1,209) = 24.38, *p* < 0.001, η^2^ = 0.10]. This confirms Hypothesis 2; according to which, a significant fall in the number of answers consistent with the misinformation was expected in the RSA group compared with the group without RSA.

In order to verify Hypothesis 3, a mediation analysis was performed. Bootstrap-generated confidence intervals were calculated to verify the existence of the mediation, as recommended by Hayes (2018). An effect is considered significant when its confidence intervals do not include zero. The PROCESS program (Hayes, 2018) was used to perform this analysis, which was performed in the group of misinformed participants.

The results indicated that a significant impact of RSA on memory confidence was found in the preliminary analysis concerning the manipulation check [*B* = 0.89, SE = 0.29, 95% CI (0.32, 1.46)]. The effect of memory confidence on yielding to misinformation was negative and significant [*B* = −0.98, SE = 0.12, 95% CI (−1.22, −0.75)]. The indirect effect of RSA on ME *via* memory confidence was also significant: [*B* = −0.87, SE = 0.29, 95% CI (−1.82, −0.34)]. This confirms Hypothesis 3, which states that memory confidence mediates the impact of RSA on ME. Interestingly, the direct effect of RSA on ME was also significant [*B* = −1.08, SE = 0.37, 95% CI (−1.82, −0.34)]. This suggests that RSA affects ME not only *via* increased memory-related self-confidence but also through some different mechanisms.

In the case of general self-confidence, its mediating effect was not statistically significant [*B* = −0.10, SE = 0.11, 95% CI (−0.35, 0.08)]. As bootstrapping is not the best method of proving the null hypothesis, quasi-Bayesian confidence intervals were also calculated by means of the *brms* package (Bürkner, 2017), running under the *R* Environment (R Core Team, 2020). The average causal mediation effect (*ACME*) was −0.10 with 95% confidence intervals: (−0.37, 0.07). This indicates a lack of a mediation effect in accordance with Hypothesis 4.

In sum, in Experiment 1, all hypotheses were confirmed: the misinformation effect and the efficacy of RSA in reducing it were replicated. Memory confidence proved to mediate the impact of RSA on ME, in congruence with existing data (Szpitalak and Polczyk, 2019b), while general confidence did not. The mediating effect of memory confidence was partial; the direct effect of RSA on ME was significant. This encourages looking for other reasons why RSA may be effective in reducing the misinformation effect, apart from the postulated and confirmed mediating effect of memory confidence.

## Experiment 2

The first three aims of Experiment 2 were similar to those of Experiment 1: replicating the misinformation effect, replicating the efficacy of RSA, and analyzing the mediating role of memory confidence and general confidence in the relationship between RSA and ME. Apart from this, analyses were performed in order to verify whether contingent self-esteem, general self-esteem, and self-efficacy moderate the impact of RSA on ME. The following hypotheses were tested:

1. The misinformation effect will be present: the number of answers consistent with the misinformation will be higher for the misinformed participants than for those in the control condition.
2. RSA will be effective: the number of answers consistent with the misinformation will be lower for the misinformed participants who undergo the RSA procedure than for those who do not.
3. Memory confidence will be a mediator of the impact of RSA on the misinformation effect.
4. General self-confidence will not be a mediator of the impact of RSA on the misinformation effect.
5. Contingent self-esteem will be a moderator of the impact of RSA on ME.
6. General self-esteem will be a moderator of the impact of RSA on ME.
7. Self-efficacy will be a moderator of the impact of RSA on ME.

### Method – Experiment 2

#### Participants

One hundred and seventy-two participants who are students at various schools were tested (125 women and 47 men). Their mean age was 17.3 years (SD = 0.79, range 16–19 years). No compensation was given for participation.

#### Materials and Procedure

The same materials and procedure for the analysis of the misinformation effect and RSA were used as in Experiment 1. In addition, the following tests were applied:

*Self-Liking – Competence Scale – Revised* (SLCS-R; Tafarodi and Swann, 2001; Polish adaptation: Szpitalak and Polczyk, 2015a). This is a 16-item questionnaire measuring two dimensions of self-esteem: self-competence (e.g., “I am a capable person”) and self-liking [e.g., “I do not have enough respect for myself” (R)]. Answers are given on a 5-point Likert scale. Higher results mean higher self-esteem and self-confidence, respectively. In the present research, the internal consistencies of both scales as measured by Cronbach alpha were 0.91 and 0.77, respectively.

*Contingent Self-Esteem Scale* (CSES; Paradise and Kernis, 1999; Polish adaptation: Szpitalak et al., 2018). This is a unidimensional questionnaire consisting of 15 items, e.g., “My overall feelings about myself are heavily influenced by how much other people like and accept me.” The questions are answered on a 5-point Likert scale, from “Not at all like me” to “Very much like me.” Higher results mean that self-esteem is more dependent on external cues. Internal consistency of this scale was 0.87.

*Generalized Self-Efficacy Scale* (GSES; Schwarzer, 1993; Polish adaptation: Juczyński, 2009). This is a tool designed to measure a general sense of perceived self-efficacy: the belief that one can perform novel or difficult tasks and cope with adversity (e.g., “I can always manage to solve difficult problems if I try hard enough”). It includes 10 items scored on a 4-point scale. Higher results mean that the person perceives them as more capable to cope effectively with tasks and problems. In this experiment, its internal consistency was 0.84.

The procedure was the same as in Experiment 1, but, instead of filler questionnaires, the above-described tools were applied. As previously, the main experimental design included two between-subjects factors: misinformation (no misinformation or misinformation present) × RSA (present or absent).

#### Results and Discussion – Experiment 2

Similarly, as in Experiment 1, the groups in which RSA was applied and the control group without it were compared as regards the results of a short questionnaire, measuring memory confidence. The mean memory confidence was significantly higher in the RSA group than in the group without RSA [*M* = 5.14, SD = 1.54 vs. *M* = 4.18, SD = 0.98; *F*(1,170) = 24.46, *p* < 0.001, η^2^ = 0.13]. This confirms the efficacy of RSA in increasing self-confidence relating to memory quality. No significant effect of RSA was present in the case of general self-confidence [*M* = 6.23, SD = 1.45 vs. *M* = 5.97, SD = 1.34; *F*(1,170) = 1.43, *p* = 0.234, η^2^ = 0.01]. This confirms the efficacy of the manipulation.

**FIGURE 2:**
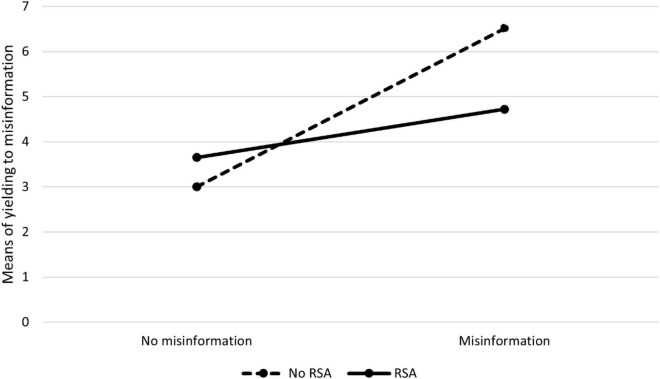
Means of yielding to misinformation across experimental conditions in Experiment 2.

Descriptive results across the experimental condition in Experiment 2 are presented in Table 2 and Figure 2.

**TABLE 2:** Means (SDs, number of participants) of yielding to misinformation across experimental conditions in Experiment 2.

RSA	No misinformation	Misinformation	Total
Absent	3.00 (1.74, 34)	6.51 (1.67, 57)	5.20 (2.40, 91)
Present	3.65 (1.84, 31)	4.72 (2.70, 50)	4.31 (2.45, 81)
Total	3.31 (1.80, 65)	5.67 (2.38, 107)	4.78 (2.46, 172)

A 2 × 2 ANOVA revealed a significant main effect of misinformation: the number of answers consistent with the misinformation was higher in the misled group than in the control group [*F*(1,168) = 49.73, *p* < 0.001, η^2^ = 0.23]. The main effect of RSA was not significant [*F*(1,168) = 3.10, *p* = 0.080, η^2^ = 0.02], but its interaction with the misinformation was significant [*F*(1,168) = 14.02, *p* < 0.001, η^2^ = 0.08]. Analysis of the simple effects revealed that the difference between the RSA and non-RSA groups was significant in the case of misled participants [*F*(1,168) = 20.01, *p* < 0.001, η^2^ = 0.11]. In the case of non-misled participants, the effect of RSA was not significant [*F*(1,168) = 1.59, *p* = 0.210, η^2^ = 0.01]. In sum, these results confirm the existence of the misinformation effect and the efficacy of RSA in reducing it.

To verify Hypothesis 3, which concerns the mediating effect of memory confidence, the same mediation analysis was performed as in Experiment 1. The impact of RSA on memory confidence was significant [*B* = 0.65, SE = 0.28, 95% CI (0.10, 1.21)], as was the negative effect of memory confidence on yielding to misinformation [*B* = −1.10, SE = 0.11, 95% CI (−1.31, −0.89)]. The indirect effect was significant [*B* = −0.72, SE = 0.33, 95% CI (−1.39, −0.08)]. This confirms Hypothesis 3. As in Experiment 1, the direct effect of RSA on yielding to misinformation was also significant [*B* = −1.07, SE = 0.31, 95% CI (−1.68, −0.46)].

In the case of general self-confidence, the mediation was not statistically significant as the bootstrap 95% confidence intervals included zero: *B* = −0.01, SE = −0.15, 95% CI (−0.29, 0.33). The ACME was <0.01 with quasi-Bayesian 95% confidence intervals (−0.32, 0.33). This confirms Hypothesis 4, which states that general self-confidence is not a significant mediator of the impact of RSA on ME.

To verify Hypothesis 5, which states that RSA would be effective mainly in the case of the participants with contingent self-esteem, a moderation analysis was performed with RSA as the predictor, CSES results as the continuous moderator, and yielding to misinformation as the dependent variable. This analysis was done only in the group of the misinformed participants and was performed by means of the PROCESS software (Hayes, 2018).

The moderating effect of CSES proved significant (*B*_*int*_ = −0.20, SE = 0.04, 95% CI (−0.27, −0.12)]. To further explore the moderation, Johnson–Neyman cut points were calculated. It turned out that the impact of RSA on ME was significant and positive from the lowest result on CSES up to the value of CSES = 38.9: the participants who underwent RSA scored higher on the ME (that is, they were more suggestible) test than those who did not. In the range of CSES from 40 points to 51, the effect of RSA was not significant. It started to be significant again from the value of CSES = 50.4 and was negative. In sum, these results indicated that, in accordance with Hypothesis 4, RSA is, indeed, helpful in the case of people with high contingent self-esteem. In the case of medium contingent self-esteem, RSA proved not useful; interestingly, in the case of low contingent self-esteem, i.e., stable self-esteem, RSA even increased the ME.

To verify the sixth hypothesis, the potential moderating effects of general self-esteem, as measured by SLCS-R, were analyzed. The moderation was not significant in the case of self-liking [*B*_*int*_ = 0.04, SE = 0.06, 95% CI (−0.08, 0.15)] and self-competence [*B*_*int*_ = 0.02, SE = 0.08, 05% CI (−0.15, 0.18)].

The seventh hypothesis concerned the moderating effect of self-efficacy. The analysis was performed in the same way as in the case of Hypothesis 6. The moderating effect of self-efficacy was not significant [*B*_*int*_ < 0.01, SE = 0.10, 95% CI (−0.19, 0.19)]; thus, the hypothesis was not confirmed.

In sum, the misinformation effect was replicated, and the efficacy of RSA was confirmed. The mediating role of memory confidence was also confirmed; in accordance with the hypothesis, general self-confidence was not a significant mediator. The effect of RSA was moderated by contingent self-esteem, but not by self-efficacy or general self-esteem.

## Experiment 3

Apart from replicating the ME and the efficacy of RSA, the main aim of Experiment 3 was to analyze the hypothesis that an important moderator of the impact of RSA on ME is feedback acceptance. As elaborated in section “Introduction,” feedback, which is not accepted, cannot be effective. Therefore, it was expected that the efficacy of RSA would be higher in the group of the participants who accepted the feedback. Apart from this, the mediating role of memory confidence was analyzed. The following hypotheses were tested:

1. The misinformation effect will be present: the number of answers consistent with misinformation will be higher for the misinformed participants than for those in the control condition.
2. The RSA will be effective: the number of answers consistent with misinformation will be lower for the participants undergoing the RSA procedure than for those who do not.
3. Memory confidence will be a mediator of the impact of RSA on the misinformation effect.
4. Feedback acceptance will be positively related to the effects of RSA on ME.

In Experiment 3, we decided to increase power to detect mediations and moderations as much as possible. As mediation and moderation analyses are only meaningful in the group of the misled participants, we decided to increase the sample size for the misled condition as much as possible and to use a smaller control group. The latter was only needed to establish the existence of the misinformation effect. Given the resources available, 452 participants were included in the misled group and 94 in the control one.

### Method – Experiment 3

#### Participants

Five hundred and forty-six participants took part in Experiment 3 – 404 women and 142 men; their mean age was 16.8 years (SD = 1.2, range: 15–31 years). Most of the participants were students at various high schools. No compensation was given for participation. Two participants failed to complete the memory confidence questionnaire.

#### Materials and Procedure

The materials, procedure for RSA, and the main experimental design were the same as in the previous experiments. In order to ensure better generalizability of this research, the original material that was used to analyze the misinformation effect was new: it was a video clip presenting a burglary and a robbery, with a duration of about 4.5 min (it was adopted from the movie “Heist” by D. Mamet). The participants were asked to watch it, without any additional information. A description of the film was presented as post-event material “in order to refresh the memory”; it included six details that were incongruent with the content of the video clip. After the post-event material, the RSA was administered in the same way as in Experiments 1 and 2, followed by a question measuring feedback acceptance: “Does your score accurately reflect your memory capabilities?” The answers were given on a 7-point Likert-like scale, from “Definitely not” to “Definitely yes.” The final memory test consisted of 12 open-ended questions, six of them relating to misled items.

#### Results – Experiment 3

As in Experiments 1 and 2, it was found that RSA, indeed, increased memory confidence; its means in the groups in which RSA was and was not applied were *M* = 4.54 (SD = 1.33) and *M* = 4.20 (SD = 0.85), respectively [*F*(1,541) = 12.46, *p* < 0.001, η^2^ = 0.02]. As an additional analysis, the correlation between feedback acceptance and memory confidence was calculated and proved significant: *r* = 0.43, *p* < 0.001. This also confirms the existence of a relationship between the efficacy of experimental manipulations and memory confidence.

**FIGURE 3:**
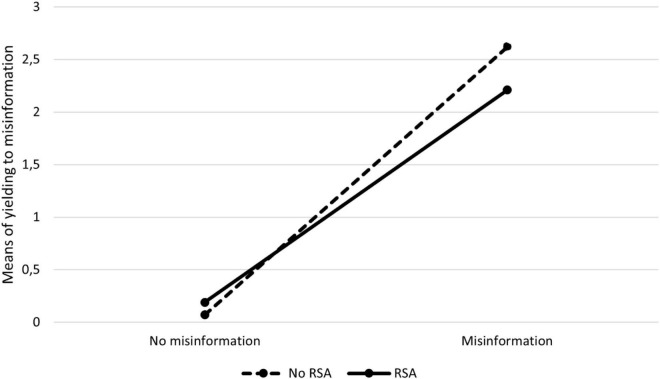
Means of yielding to misinformation across experimental conditions in Experiment 3.

Descriptive results in all experimental conditions are presented in Table 3 and Figure 3.

**TABLE 3:** Means (SDs, number of participants) of yielding to misinformation across experimental conditions in Experiment 3.

RSA	No misinformation	Misinformation	Total
Absent	0.07 (0.25, 62)	2.62 (1.73, 222)	2.06 (1.86, 284)
Present	0.19 (0.40, 32)	2.21 (1.29, 230)	1.97 (1.38, 262)
Total	0.11 (0.31, 94)	2.41 (1.53, 452)	2.02 (1.65, 546)

The general effect of misinformation was significant and large [*F*(1,542) = 193.63, *p* < 0.001, η^2^ = 0.26]. The general effect of RSA and its interaction with the misinformation were not significant [*F*(1,542) = 0.75, *p* = 0.386, η^2^ < 0.01 and *F*(1,542) = 2.61, *p* = 0.107, η^2^ < 0.01, respectively]. However, as the hypothesis concerning RSA only applies to misinformed people, planned comparisons were more appropriate. As in Experiments 1 and 2, these comparisons revealed that the participants in the RSA subgroup of the misled group yielded to misinformation significantly less than those in the subgroup without RSA [*F*(1,542) = 9.78, *p* = 0.002, η^2^ = 0.02]. In the non-misinformed group, the difference between the participants who were and were not exposed to RSA was not significant [*F*(1,542) = 0.17, *p* = 0.684, η^2^ < 0.01]. However, the misinformation effect was present both in the group without RSA [*F*(1,542) = 164.30, *p* < 0.001, η^2^ = 0.23] and with RSA [*F*(1,542) = 59.76, *p* < 0.001, η^2^ = 0.10]; although, in the latter case, it was smaller.

In the group of the misinformed participants, the mediation effect of memory confidence was significant [*B* = −0.17, SE = 0.05, 95% CI (−0.27, −0.06)]. As in the previous experiments, RSA increased memory confidence [*B* = 0.35, SE = 0.11, 95% CI (0.13, 0.56)]. Memory confidence reduced yielding to misinformation [*B* = −0.48, SE = 0.06, 95% CI (0.59, −0.37)]. The direct effect of RSA on ME was not significant [*B* = −0.25, SE = 0.14, 95% CI (0.51, 0.02)].

Hypothesis 4 postulated that RSA is effective when feedback is accepted. Feedback acceptance could only be scored in the group with RSA; therefore, no moderation analysis that included RSA was possible, and Hypothesis 4 was analyzed in the group of the misinformed participants who underwent the RSA procedure by means of computing the correlation between the level of feedback acceptance and yielding to misinformation. Notably, there was considerable variance in the measure of feedback acceptance. The answers to the question “Does your score accurately reflect your memory capabilities” were given on a 7-point scale with the following frequencies: 1 (definitely not) –5.4%; 2–7.2%; 3–12.5%; 4–23.7%; 5–36.2%; 6–11.8%; 7 (definitely yes) –3.2%. The results of the correlational analysis confirmed the hypothesis: Pearson’s *r* was −0.54 (*p* < 0.001), which indicates that the higher the feedback acceptance, the lower the yielding to misinformation.

In sum, all four hypotheses tested in Experiment 3 were confirmed. However, a caveat is needed here: the lack of interaction between the factors: misinformation and RSA mean that the efficacy of RSA is not certain here, even if the analysis of simple effects confirms this efficacy.

## General Discussion

The main aim of the three experiments presented in this paper was to present further data on RSA, which is a method of reducing the tendency to rely on misinformation when giving memory reports. This tendency, known as the memory misinformation effect, was present in all three experiments. This confirms the robustness and replicability of this effect. This is a warning for justice systems, as the misinformation effect may be an important cause of incorrect testimonies and their consequences.

Not many methods of reducing the misinformation effect have been described. The method presented in this paper, namely, RSA, is intended for witnesses who, in fact, do remember the correct original information yet prefer to rely on external sources even if the information stemming from them contradicts the original information. It was assumed that the reason for such behavior is lack of confidence in one’s own memory. Therefore, RSA aims to increase memory confidence. It proved effective in all three experiments described in the present study. This is a replication of numerous existing studies on its efficacy (Szpitalak, 2012, 2015; Szpitalak and Polczyk, 2013, 2015b,2019a,b). RSA may be a promising way to develop techniques that are suitable for use in the context of real interrogations.

To be exact, in Experiments 1 and 2, the efficacy of RSA was proved both in the light of its significant interactions with misinformation and simple effects, while, in Experiment 3, the interaction was not significant, although appropriate planned comparisons were significant and consistent with the hypothesis. In Experiment 3, the main original material was different from Experiments 1 and 2. The change was applied in order to ensure better generalizability of the results but may also explain the slightly different results concerning RSA. Recall that the main hypothesis stated that RSA is effective mainly among persons who do realize the differences between the original and postevent materials. Perhaps, there were less such participants due to the change of materials.

In the present study, some possible mediators and moderators of the impact of RSA on ME were studied. First of all, it was assumed that memory confidence would mediate the effect of RSA; this was confirmed in all three experiments and is congruent with other existing data (Szpitalak and Polczyk, 2019b). This was the core hypothesis: RSA should increase confidence in one’s own memories and, therefore, increase the tendency to rely on them instead of other sources of information. Obviously, this reasoning assumes that some participants remember both the original and the misleading post-event information. As mentioned in section “Introduction,” there are now sufficient empirical data to assume so (Blank, 1998; Szpitalak and Polczyk, 2010; Polczyk, 2017). Interestingly, RSA has already been shown to be effective, particularly among persons who are aware of discrepancies between original and post-event information (Szpitalak and Polczyk, 2015b, Experiment 2).

Apart from acting *via* memory confidence, RSA showed a direct effect on ME. This result should be treated with caution as it only appeared in two out of the three analyses. Nevertheless, apart from mechanisms, which consist in increasing self-confidence, the result encourages considering other possible mechanisms of RSA. As described in section “Introduction,” high self-confidence is beneficial in a wide range of situations. For example, it is possible that it encourages more careful and scrupulous searching of memory. This should be analyzed in further research.

As for moderators, it was hypothesized (and successfully shown) that self-esteem matters as regards the efficacy of RSA. To be exact, our hypothesis concerned both contingent self-esteems, i.e., self-esteem that is highly dependent on external confirmation and general self-esteem. It was assumed that RSA would not be effective among people with stable self-esteem as they are not dependent on and do not need constant confirmation of their value. This hypothesis was confirmed. In contrast, it turned out that general self-esteem was not important for the efficacy of RSA. Overall, this is in agreement with views assuming that self-esteem is not a unitary trait and having generally high self-esteem does not necessarily generalize to all areas and abilities (e.g., Coopersmith, 1967; O’Brien and Epstein, 1988). Similarly, it was also hypothesized that the sense of self-efficacy would have similar effects: the participants with a high sense of self-efficacy might benefit from RSA less than those with lower self-efficacy because they are less dependent on external confirmations of this efficacy. This hypothesis was also not confirmed. One possible reason for this may be the fact that the tool used to measure self-efficacy, GSES (Schwarzer, 1993), was intended to measure a general trait. Such broad self-estimations may not necessarily generalize to specific abilities. It is possible that a tool measuring specific self-efficacy related to memory would moderate the impact of RSA on ME.

Another moderator of the effects of RSA on ME was feedback acceptance. It was hypothesized that, if positive feedback concerning memory is not accepted, memory-related self-confidence would not be enhanced, and the dependency on misleading information would not be reduced. This hypothesis was confirmed. In addition, our results confirm that feedback is not accepted and incorporated automatically. Apparently, it was interpreted and processed cognitively. It is possible, although our data cannot confirm this, that people treat feedback as valid only if it is congruent with their conceptions of self, at least to some degree (Markus, 1977; Swann, 1987). As Esses (1989) suggested, it may also be the case that feedback is accepted when its affective tone matches a mood state of an individual. After receiving the feedback, a person searches his or her memory to obtain evidence that confirms or discredits the content of the feedback. In a situation in which the memory that confirms the content of the feedback is activated, the person accepts the feedback and is willing to modify his or her self-image in line with the feedback. In a situation in which the subject does not find confirmation for the content of the received feedback, he or she usually rejects its content (Swann, 1987). However, memories that support both negative and positive feedback are usually available (Esses, 1989); in which case, the mood of the person seems to play a key role.

Finally, potential problems with our study should be mentioned. It should be acknowledged the RSA, in general, may not be free from some risks, stemming exactly from increased confidence of a witness. The relationship between confidence and accuracy is complicated (Olsson, 2000; Kebbell, 2009). Increased confidence may be dangerous if a witness has an inaccurate recollection.

Also, another caveat is worth mentioning. As elaborated in section “Introduction,” RSA is expected to be effective mainly among persons who are aware of the discrepancies between the original and post-event information. But we also speculated that warning against discrepancies between both sources is effective among witnesses who are aware of the discrepancies between them. It may be that RSA would be, in a way, redundant with warning in the case of such witnesses.

Furthermore, witnesses who are confident in the quality of their memory would probably benefit from it less.

Also, we are aware that RSA in its present form is of little use for forensic practitioners. It is certainly impossible to provide a real witness with fake positive feedback; this would be impossible for ethical and, probably, also for legal reasons. Having a witness write down his or her greatest achievements in life would also be strange. The present research is, therefore, basic in its nature but can, nevertheless, inspire development of a technique that is suitable for real forensic settings. Efforts to construct such a method are currently in progress.

There may be a problem with the measurement of feedback acceptance. It consisted in asking a question: “Does your score accurately reflect your memory capabilities?” It is possible that a participant may feel that his or her memory was not good but chose to accept the feedback due to perceiving the experiment to be correct.

In Experiments 1 and 2, the final memory test required the participants to make forced responses, without the possibility to refuse an answer or to indicate uncertainty. This is a possible limitation of the present study as, in reality, witnesses normally are (or should be) asked little questions in the form of closed alternatives and are allowed and encouraged to state if they are unsure about certain information.

## Data Availability Statement

The original contributions presented in the study are included in the article/Supplementary Material, further inquiries can be directed to the corresponding author/s.

## Ethics Statement

The studies involving human participants were reviewed and approved by the Research Ethics Committee at the Institute of Psychology of the Jagiellonian University; Decision Nr KE/01/092018. Written informed consent from the participants’ legal guardian/next of kin was not required to participate in this study in accordance with the national legislation and the institutional requirements.

## Author Contributions

MS: conceptualization, empirical research, and writing text. RP: statistical analysis and writing text. Both authors contributed to the article and approved the submitted version.

## Conflict of Interest

The authors declare that the research was conducted in the absence of any commercial or financial relationships that could be construed as a potential conflict of interest.

## Publisher’s Note

All claims expressed in this article are solely those of the authors and do not necessarily represent those of their affiliated organizations, or those of the publisher, the editors and the reviewers. Any product that may be evaluated in this article, or claim that may be made by its manufacturer, is not guaranteed or endorsed by the publisher.
